# Nanomaterial-Driven Precision Immunomodulation: A New Paradigm in Therapeutic Interventions

**DOI:** 10.3390/cancers16112030

**Published:** 2024-05-27

**Authors:** Alaa A. A. Aljabali, Mohammad A. Obeid, Omar Gammoh, Mohamed El-Tanani, Vijay Mishra, Yachana Mishra, Sumedha Kapre, Sushesh Srivatsa Palakurthi, Sk. Sarif Hassan, Debaleena Nawn, Kenneth Lundstrom, Altijana Hromić-Jahjefendić, Ángel Serrano-Aroca, Elrashdy M. Redwan, Vladimir N. Uversky, Murtaza M. Tambuwala

**Affiliations:** 1 Faculty of Pharmacy, Department of Pharmaceutics & Pharmaceutical Technology, Yarmouk University, Irbid 21163, Jordan; alaaj@yu.edu.jo (A.A.A.A.); m.obeid@yu.edu.jo (M.A.O.); 2 Department of Clinical Pharmacy and Pharmacy Practice, Faculty of Pharmacy, Yarmouk University, Irbid 21163, Jordan; omar.gammoh@yu.edu.jo; 3 College of Pharmacy, Ras Al Khaimah Medical and Health Sciences University, Ras Al Khaimah P.O. Box 11172, United Arab Emirates; eltanani@rakmhsu.ac.ae; 4 School of Pharmaceutical Sciences, Lovely Professional University, Phagwara 144411, Punjab, India; vijaymishra2@gmail.com; 5 School of Bioengineering and Biosciences, Lovely Professional University, Phagwara 144411, Punjab, India; yachanamishra@gmail.com; 6 Department of Pharmaceutical Sciences, Irma Lerma Rangel School of Pharmacy, Texas A&M University, Kingsville, TX 78363, USA; sumedhakapre@gmail.com (S.K.); susheshsrivatsa98@gmail.com (S.S.P.); 7 Department of Mathematics, Pingla Thana Mahavidyalaya, Maligram, Paschim Medinipur 721140, West Bengal, India; sksarifhassan@pinglacollege.ac.in; 8 Indian Research Institute for Integrated Medicine (IRIIM), Unsani, Howrah 711302, West Bengal, India; debaleena.nawn@gmail.com; 9 PanTherapeutics, CH1095 Lutry, Switzerland; lundstromkenneth@gmail.com; 10 Department of Genetics and Bioengineering, Faculty of Engineering and Natural Sciences, International University of Sarajevo, Hrasnicka Cesta 15, 71000 Sarajevo, Bosnia and Herzegovina; ahromic@ius.edu.ba; 11 Biomaterials and Bioengineering Lab, Centro de Investigación Traslacional San Alberto Magno, Universidad Católica de Valencia San Vicente Mártir, c/Guillem de Castro 94, 46001 Valencia, Spain; angel.serrano@ucv.es; 12 Department of Biological Science, Faculty of Science, King Abdulaziz University, P.O. Box 80203, Jeddah 21589, Saudi Arabia; lradwan@kau.edu.sa; 13 Centre of Excellence in Bionanoscience Research, King Abdulaziz University, Jeddah 21589, Saudi Arabia; 14 Therapeutic and Protective Proteins Laboratory, Protein Research Department, Genetic Engineering and Biotechnology Research Institute, City for Scientific Research and Technology Applications, New Borg EL-Arab, Alexandria 21934, Egypt; 15 Department of Molecular Medicine, Morsani College of Medicine, University of South Florida, Tampa, FL 33612, USA

**Keywords:** immunotherapy, nanomaterials, drug delivery, immune modulation, cancer vaccines, personalized immunotherapy

## Abstract

**Simple Summary:**

This review assesses the integration of nanotechnology and immunotherapy, with a specific focus on the utilization of nanomaterials to modulate the immune system in conditions such as cancer and autoimmunity. Liposomes, polymers, and inorganic nanoparticles (NPs) are versatile nanomaterials capable of effectively transporting immunomodulatory molecules. Their interactions with immune cells have contributed to the development of checkpoint inhibitors, improved cancer vaccines, and the optimization of adoptive cell therapies. These approaches enable the fine-tuning of immune responses with minimal adverse effects. Positioned at the forefront of the convergence of nanotechnology and immunology, nanomaterial-based platforms have the potential to revolutionize patient-centered immunotherapy. These systems are used in the transformative era of disease management by prioritizing safety, personalization, and compliance with regulations.

**Abstract:**

Immunotherapy is a rapidly advancing field of research in the treatment of conditions such as cancer and autoimmunity. Nanomaterials can be designed for immune system manipulation, with precise targeted delivery and improved immunomodulatory efficacy. Here, we elaborate on various strategies using nanomaterials, including liposomes, polymers, and inorganic NPs, and discuss their detailed design intricacies, mechanisms, and applications, including the current regulatory issues. This type of nanomaterial design for targeting specific immune cells or tissues and controlling release kinetics could push current technological frontiers and provide new and innovative solutions for immune-related disorders and diseases without off-target effects. These materials enable targeted interactions with immune cells, thereby enhancing the effectiveness of checkpoint inhibitors, cancer vaccines, and adoptive cell therapies. Moreover, they allow for fine-tuning of immune responses while minimizing side effects. At the intersection of nanotechnology and immunology, nanomaterial-based platforms have immense potential to revolutionize patient-centered immunotherapy and reshape disease management. By prioritizing safety, customization, and compliance with regulatory standards, these systems can make significant contributions to precision medicine, thereby significantly impacting the healthcare landscape.

## 1. Introduction

Immunomodulatory drug delivery systems (IDDSs) are innovative smart nanocarriers that are intrinsically utilized for the targeted and controlled modulation of the immune system. Immunomodulating agents such as cytokines, antibodies, and vaccines are employed to enhance efficacy and safety while minimizing adverse effects. This is achieved by delivering these agents directly to immune cells or target tissue [1,2,3]. Immunomodulatory DDSs have demonstrated significant efficacy in a wide range of diseases including cancer, autoimmune disorders, and infectious diseases. Specifically, it enables the direct delivery of therapeutic agents to cancer cells, promotes tumor response, and reduces the harmful side effects of immunotherapy drugs for cancer treatment [4]. IDDSs have also shown promising results by effectively delivering drugs for the treatment of rheumatoid arthritis (RA) [5]. As IDDSs are still in the early stages of development, they offer numerous advantages for drug delivery compared to traditional methods. Only a small volume of the drug is required, allowing local administration at therapeutic dosages. As a result, systemic toxicity is minimized, either because of the inability to reach therapeutic concentrations systemically, or the ability to use lower drug doses. This targeted approach enhances drug effectiveness by precisely targeting immune cells or tissues [6,7], while minimizing adverse effects, thus improving the risk–benefit ratio [6]. Moreover, IDDSs may improve patient compliance and adherence to treatment regimens [8]. 

IDDS represents an inaugural discipline within the field of pharmaceutical research that unites immunology with drug delivery applications to develop groundbreaking therapeutic strategies [9]. By precisely modulating immune responses, it has the potential to improve treatment effectiveness and overcome the constraints associated with conventional interventions to fine-tune the immune responses in a targeted manner. This innovative breakthrough has generated heightened enthusiasm for harnessing our understanding of the immune system for therapeutics. In alignment with this objective, IDDSs leverage the manipulation of the immune system to attain augmented treatment efficacy [3,10]. 

Additional methods of drug delivery involve the use of NP-based drug carriers, specifically, liposomes or polymeric NPs. These carriers were specifically designed to facilitate targeted delivery, allowing precise control over drug encapsulation and release. This targeted approach minimizes off-target effects and maximizes therapeutic effectiveness [11,12]. Furthermore, the utilization of immunotherapeutic strategies in conjunction with IDDSs presents a promising solution for overcoming resistance to cancer therapy and counteracting the immune-suppressing mechanisms associated with cancer [13,14]. As a result, these advancements offer hope that extends beyond the limitations currently faced in the field of immunotherapy [15,16] thereby overcoming obstacles to immunotherapy potential [17,18].

However, the development of IDDSs poses challenges in maintaining immune modulation without causing significant disruption, or in understanding the immune regulation and associated effects of these immunomodulatory agents [17]. The potential of IDDSs relies on overcoming challenges in material science, comprehending immunological aspects, understanding immune regulation, and understanding the impact of immunomodulatory agents [18]. The term IDDS encompasses a shared space in the fields of immunology and pharmaceutical innovation, where researchers and clinicians collaborate to explore the future possibilities of therapeutics [11,19]. In this context, IDDSs involve the utilization of nanocarriers, such as synthetic microparticles, liposomes, and chitosan, to enhance the body’s response to immunization [20]. Therefore, integrating the technical aspects of drug delivery into cancer immunotherapy holds great potential for introducing a new era of safe and effective treatment approaches that offer novel combinations of treatment and personalized cancer immunotherapy [21]. 

Combinatorial immunotherapy, such as chemotherapy and immunotherapy, acts synergistically to potentiate antitumor immune responses. Mechanistically, chemotherapy works through direct cytotoxic effects, induction of immunogenic cell death, and modulation of immune regulatory pathways, thus complementing the enhancement of antitumor immune responses via immunotherapy [22]. Clinical observations have shown improved response and survival rates for some cancers. Dose and scheduling issues are paramount for balancing the maximal efficacy, minimal toxicity, and immune-related adverse events. Similarly, combining phototherapy with immunotherapy, such as photodynamic therapy (PDT) and photothermal therapy (PTT), increases immune activation through activation of tumor-specific and systemic antitumor responses [22]. The optimization of treatment parameters and the identification of predictive biomarkers for patient selection are challenging. Other combinatorial approaches include radiation therapy, targeted therapy, and cytokine therapy with immunotherapy, and all possess unique synergistic interactions that can improve cancer treatment outcomes [22]. Such approaches represent one of the most exciting opportunities for boosting the efficacy of combinatorial immunotherapies by acting effectively as carriers of therapeutic agents with targeted delivery at the tumor site. The tunable characteristics of mesoporous silica NPs make them attractive candidates for the optimization of treatment parameters and help overcome related challenges in combinatorial immunotherapy, allowing improvement in cancer treatment.

In summary, IDDSs represent a promising approach to immunomodulatory drug delivery, providing precise targeting, improved efficacy, and enhanced safety. However, ongoing research is essential to fully unlock the potential of revolutionizing disease treatment strategies [23,24]. The safety concerns regarding nanomaterials in immunotherapy are principally related to their unique properties and interactions with biological systems. Biocompatibility is the single most important factor for addressing negative reactions, and knowledge of biodistribution helps understand the prevention of toxicity. Assessment of immunogenicity is critical and long-term safety evaluations are required to identify possible risks. Environmental impact and the possibility of meeting regulatory requirements are other factors of general concern. In general, these methods require full preclinical characterization, extensive toxicity testing, and active safety monitoring in clinical trials [25,26]. Each type of nanomaterial has advantages and disadvantages that dictate the preference for one type over another, as presented in Table 1.

The present review outlines the exciting and fast- progressing IDDS field using nanomaterials for the precise and targeted modulation of the immune system. Herein, we describe the detailed interplay between nanomaterials and immune cells, which is considered a prerequisite for tailored modulation of immune responses in therapeutic applications. This study aimed to correlate the properties of nanomaterials with their specific applications in diseases, such as cancer, autoimmunity, and allergy, thus providing insight into the innovative potential of nanotechnology in immunotherapy. Nanomaterials selected for these diseases should satisfy certain criteria to meet the efficacy and specificity of therapeutic applications. Nanomaterials intended for targeting cancer cells typically have features designed to ensure their selective accumulation in tumors along with an adequate structure or composition that reduces off-target effects. These approaches vary from surface modifications that improve tumor cell homing and penetration, to payloads that can elicit cytotoxic effects once internalized within cells [27]. Nanomaterials have been used to regulate immune responses in autoimmunity, which requires accurate immunomodulatory properties that can regulate aberrant immune reactions. These materials are often used for surface functionalization to permit interaction with immune cells for the delivery of immunosuppressive agents or antigens to induce tolerance. Nanomaterials can be used to target key players in the allergic cascade to prevent or reduce hypersensitivity responses [28]. Typical examples of such applications include liposomes for the encapsulation of drugs targeted specifically to cancer cells, or for carrying immunomodulatory agents for autoimmunity. These therapeutic strategies include engineering polymeric NPs for the controlled release of allergen-specific treatments. Ultimately, nanomaterials are selected for their designed features, such as size, surface chemistry, and payload capacity, to optimize the therapeutic outcomes in the unique pathophysiology of each disease state. Engineered nanomaterials can be tailored to specifically interact with immune cell receptors or signaling pathways responsible for allergic reactions to modulate immune activation and subsequently diminish allergic responses. For example, NPs such as liposomes or polymeric NPs can be used to encapsulate allergens or immunomodulators such that they can be selectively delivered to immune cells at allergic inflammatory sites [29]. For example, advanced nanotechnologies are aimed at harnessing nanomaterials in allergen desensitization strategies, including allergen-specific immunotherapy (AIT). NPs can be designed based on the shape of the allergen structure or loaded with an allergen peptide for controlled delivery, which induces immune tolerance upon administration to reduce allergic sensitization over time. Nanomaterials may also be useful in designing novel strategies to modulate immune responses to prevent allergies. This is achieved through immune activation by locally delivering anti-inflammatory agents or immunomodulators to regulate immune cell activation. Using the distinctive properties of nanomaterials, the treatment of allergies has been revolutionized providing personalized and effective management of several different types of allergic diseases [30,31]. Table 2 summarizes a comparison of currently available immunotherapy paradigms.

In the present study, we conducted a thorough SWOT analysis of nanomaterial-based IDDSs. These systems combine nanotechnology with immunology, and have versatile applications in precision medicine (Table 3). This analysis highlights the advantages of these systems, including precise targeting and enhanced efficacy through controlled release of immunomodulatory agents. However, it also acknowledges the challenges in their design and potential toxicity concerns, emphasizing the need for safety assessment. This study also discusses promising prospects such as personalized medicine, interdisciplinary collaborations, and combination therapies. However, it is important to consider potential threats such as regulatory obstacles, manufacturing complexities, and immunogenicity. By considering both internal and external factors, this study emphasizes the transformative potential of nanomaterial-based systems in revolutionizing immunotherapy and disease treatment strategies.

## 2. Immunomodulatory Strategies in Drug Delivery 

The most important parameter for optimizing the design of IDDSs for molecular systems and receptors is the addition of surface ligands or antibodies, thereby showing selective interaction with molecular markers or receptors expressed on the surface of target cells [32]. It has the potential to contribute to an increase in the specificity of targeting and improvement in cellular uptake. Furthermore, biocompatibility ensures that the IDDS is minimally toxic and friendly to biological systems, and the controlled release mechanisms optimize the modulation of drug concentrations at the target site to yield the maximum therapeutic outcome [33]. Stability under physiological conditions ensures structural integrity and drug encapsulation within the IDDS, which circulates in the body without premature drug release or degradation [34]. Furthermore, the size and shape of IDDS considerably affect cellular uptake and biodistribution. In most instances, IDDSs at the nanoscale have better penetration across biological barriers and cellular internalization. Charged surface properties, such as zeta potential, equally play a role in affecting the stability, circulation time, and binding of IDDSs to target cells, and critically influence the in vitro and in vivo characteristics of IDDSs. The degradability of IDDS in a controlled manner after drug release ensures minimal long-term accumulation and potential toxicity. Such characteristics can be incorporated into the design of IDDSs so that effective, specific, and efficient IDDSs can be designed for various applications [35,36].

### 2.1. Targeting Immune Cells 

Current innovations in this new field of combinatorial pharmacological interventions, along with immune responses, are ongoing, particularly to enhance drug delivery precision [12]. Over time, there has been increasing interest in the development of intracellular drug delivery systems targeting immune cells with acumen [37]. Such an IDDS, upon interaction with immune cells, can modulate immune responses at the cellular level for more effective delivery of therapeutic modalities. Another necessary factor for the successful implementation of an IDDS is immune cell tracking. For instance, nanocarriers such as liposomes or polymeric NPs have been engineered to incorporate therapeutic agents [38,39]. Thus, with the specificity of targeting immune cells and incorporating therapeutic agents within nanocarriers, it has been postulated that IDDSs offers a promising approach for treating immune-related disorders and diseases [18]. 

**Figure 1:**
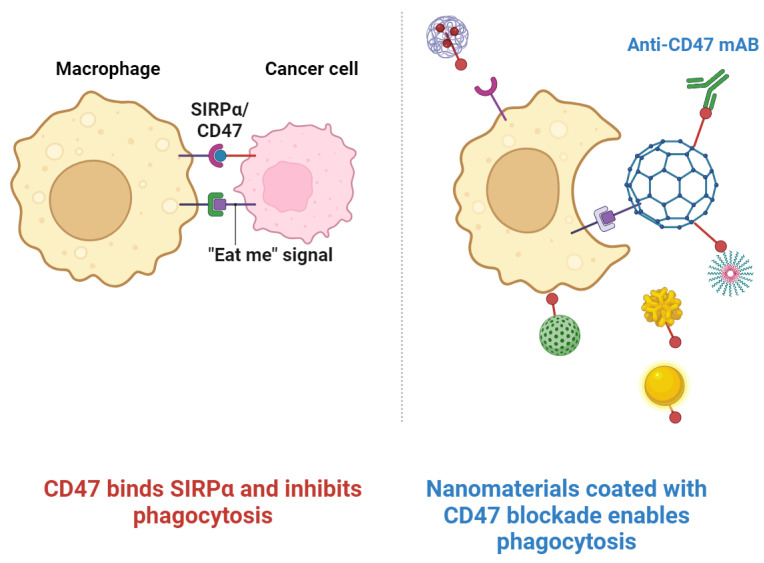
The interaction between CD47 and SIRP is crucial for maintaining homeostasis of the immune system. CD47 binds to SIRP, sends a “do not eat me” signal to macrophages, and inhibits phagocytosis. The blockade of this interaction by anti-CD47 monoclonal antibodies allows macrophages to phagocytize cancer cells. Anti-CD47 mAbs encapsulated in nanomaterials can thus be used to ensure that their delivery is targeted and is more efficient in inhibiting the interaction between CD47 and SIRP. This represents a bright milestone in the development of cancer immunotherapies. Images were acquired using Biorender.com software.

Among the antigen-presenting cells in the immune system, dendritic cells (DCs) are particularly significant as they capture antigens and activate T cells, thus initiating immune responses [40]. Targeting DCs can direct specific immune reactions. Nanocarriers coated with ligands that recognize DC-specific markers, such as CD11c and CD205, facilitate the uptake of immunomodulatory substances by DCs as shown in Figure 1. These interactions trigger antigen presentation and T-cell activation, subsequently regulating immune processes [41,42]. Targeted delivery has proven successful in generating enhanced immune responses against pathogens and cancers [43].

Furthermore, macrophages can be categorized into two distinct functional states, M1 and M2, which respond differently to various stimuli, including specific cytokines and cellular mediators [44]. Macrophages are crucial for both inflamed tissues and tissue homeostasis. Nanocarriers designed to deliver medications are equipped with specific ligands that recognize surface markers on macrophages, such as CD64 and CD206. This targeted delivery ensures precise localization and action at the desired site, allowing immunomodulatory drugs to reverse inflammation through immune evasion strategies and induce tissue remodeling [45,46]. Nanocarriers, including liposomes, polymeric NPs, and mesoporous silica NPs, can be designed for targeted drug delivery to macrophages and T-cells (Figure 1). The surfaces of nanocarriers can be functionalized with ligands that specifically recognize surface markers overexpressed in immune cells, such as CD64 and CD206 for macrophages and CD3 and CD28 for T-cells. Functionalization with such ligands ensures that the nanocarriers, at a minimum, bind selectively to target immune cells, to localize and release drugs at the site of interest [47]. The mechanism of action involves targeted delivery of immunomodulatory drugs to immune cells via nanocarriers. However, drug targeting is non-specific. Targeted delivery of drugs to immune cells through nanocarriers brings the drug to the targeted site, evoking immune modulation and resulting therapeutic response. Off-target effects and systemic exposure are minimized when drug targeting, as in this example, is designed to elicit a greater therapeutic effect. The nanocarrier acts as a vehicle, bringing drugs to immune cells, and the drugs exert their effects at the targeted site, evoking immune modulation, and resulting in therapeutic responses. Research has also revealed that some nanocarriers can act as drug delivery systems for immune cell targeting. For instance, Majumder and Minko [47] elaborated on the benefits of developing a nanocarrier-based delivery system as a remedy, bypassing conventional drug dosage formulation, with a special focus on targeted delivery at the disease site. For example, Elzoghby et al. illustrated the use of nanocarriers functionalized with lactoferrin for tumor-targeted drug delivery, demonstrating the potential of nanocarriers in targeted therapy. In essence, delivery to immune cells, such as macrophages and T-cells, is made possible using nanocarriers [48]. Appropriate modification of nanocarrier surfaces with specific ligands can provide immune cells with high target selectivity, leading to effective immunomodulatory drug delivery that modulates immune responses. The importance and efficiency of nanocarriers in achieving drug delivery for immunomodulation have been emphasized in these studies.

**Figure 2:**
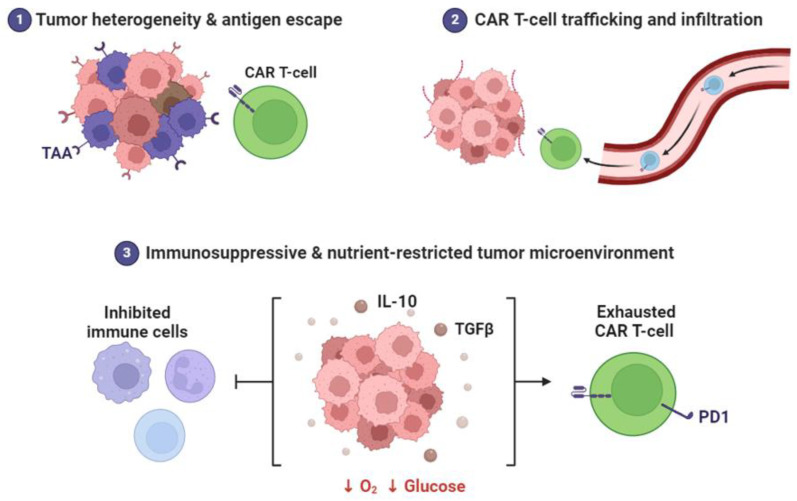
Tumor heterogeneity and antigen escape in CAR T cell therapy. CAR T cells navigate the tumor microenvironment, including the immunosuppressive milieu characterized by IL-10 and TGF secretion. This leads to reduced oxygen levels, glucose depletion, immune cell inhibition, and CAR-T-cell exhaustion. The images were generated using Biorender.com software.

T-cells play a crucial role in adaptive immunity by orchestrating the immune response against pathogens and neoplasms. They can be engineered to provide therapeutic benefits through immune checkpoint blockade or CAR T cell therapy (Figure 2) [49,50]. Nanocarriers decorated with modified ligands that target T cell receptors and co-stimulating molecules, such as CD3 and CD28, enable specific delivery of their contents [51]. This targeted delivery enhances T-cell activation, proliferation, and cytokine production, thereby enhancing the effectiveness of immunotherapy [18]. Nanomaterials possess desirable properties, such as chemical stability and inertness, which make them suitable carriers for delivering drugs to immune cells. For instance, liposomes coated with CD11c antibodies and loaded with antigens can specifically reach DCs [52,53]. Liposomes have been designed to deliver drugs to antigen-presenting dendritic cells (DCs). Chemical stability and inertness are the advantages of liposomes; therefore, they can be used as carriers for drug delivery to immune cells. Consequently, liposomes protect drugs against degradation, attain controlled release, enhance pharmacokinetic ability, and, most importantly, reach the desired immune cells via passive targeting [54]. This is based on the design of liposomes coated with CD11c antibodies that interact with the CD11c/CD18 integrins on the surface of DCs. Surface modification of liposomes has been proven to allow the specific recognition and conjugation of liposomes to DCs for specific drug delivery to immune cells [55]. Its mechanism of action involves receptor-mediated endocytosis, in which DCs internalize antigens loaded into liposomes and are coated with CD11c antibodies. Inside the cells, antigens released by liposomes are processed for presentation to T-cells, resulting in the induction of an immune response. This delivery specificity ensures that antigens reach DCs directly, thus inducing immune responses specific to the intended antigens [55]. Some studies have shown that the potential of immunoliposomes for targeted drug delivery occurs through selective delivery of encapsulated drugs to cells via the interaction of cell surface proteins using liposomes modified with antibodies [56]. Subsequent studies have dealt with more specifically targeted, drug-loaded liposomes functionalized with monoclonal antibodies (mAbs) to target antigens expressed on the cell surface, demonstrating the flexibility and specificity of antibody-coated liposomes for drug delivery [57].

In various disease models, targeted delivery of immunomodulators to M2 macrophages has been achieved using mesoporous silica NPs functionalized with mannose ligands. Engineered NPs carrying encapsulated immunomodulatory cytokines have also been explored for T-cell-targeted immunotherapies with the potential for further development in this field [58].

The field of immunomodulatory drug delivery has entered a new phase called precision medicine, in which targeted interventions using immune-cell-targeting techniques have become possible, as presented in Table 4. The fusion of nanotechnology and immunology provides researchers with tools to induce immune responses for therapeutic purposes [59]. Immune-cell-targeting techniques have allowed immunomodulatory drug delivery for potential precision medicines, with the increasing feasibility of targeted interventions. The strategy is oriented toward individual patients with specific genetic makeup, lifestyle, and environment. The immune-cell-targeting permits precise control of the immune response and outcome of therapy [60,61].

However, it is essential to refine targeting strategies, optimize nanocarrier design, and establish the clinical efficacy of IDDSs. This requires a thorough understanding of immune cell biology, ligand–receptor interactions, and immune cascades [62]. Through a combination of system immunology and advanced imaging techniques, the complexities of interactions between immune cells can be uncovered, leading to the development of more effective and safer IDDSs. Collaboration across different disciplines will play a crucial role in translating these advancements into tangible clinical applications, ultimately paving the way for personalized immunomodulation. Imaging plays an important role in immunotherapy [63] by encapsulating imaging agents, such as fluorescent dyes or contrast agents, for different imaging modalities, such as magnetic resonance imaging, computed tomography imaging, positron emission tomography, and optical imaging [64]. Additionally, these techniques enable the in vivo visualization and monitoring of nanomaterial distribution, accumulation, and clearance following administration to ensure effective targeted delivery at the tumor site with minimum off-target effects [65]. Moreover, imaging techniques allow the real-time assessment of therapeutic efficacy because of insights into the interactions of nanomaterials with the immune system and their effects on tumor growth and immune responses. This allows for early detection of response or resistance to treatment, enabling treatment decisions and timely adjustment of therapy regimens [66].

**Table 4:** Nanomaterial approaches for targeting immune cells during drug delivery present different categories of nanocarriers that can be used to target specific immune cell populations.

Nanocarrier Type	Targeted Immune Cells	Surface Ligands/Antibodies/Peptides	Functional Outcome	Examples of Nanomaterial-Based Systems	References
Liposomes	Dendritic Cells	Mannose Receptors	Enhanced Antigen Presentation	Liposomes loaded with tumor antigens and CD40 ligands	[67,68]
Polymeric NPs	Macrophages	CD47-SIRPα Interactions	Inhibition of Phagocytosis	Polymer-based NPs with CD47 for macrophage evasion	[69,70]
	Macrophages	LPS Mimics	Enhanced Immune Activation	Polymer-based NPs with LPS mimics for macrophages	[71,72,73]
	Monocytes	Anti-inflammatory Cytokines	Repolarization of Monocytes	Polymer-based NPs delivering IL-10	[74,75]
Lipid NPs	T-Cells	T Cell Receptor Ligands	T Cell Activation	Lipid NPs coated with TCR ligands	[76,77]
	Regulatory T-Cells	TGF-β Receptor Blockade	Suppression of Treg Functionality	Lipid-based NPs with TGF-β receptor inhibitors	[78,79]
	Neutrophils	CXCR2 Ligands	Neutrophil Chemotaxis Inhibition	Lipid NPs with CXCR2 ligands	[80,81]
Gold NPs	Natural Killer Cells	Natural Cytotoxicity Receptors	Increased Cytotoxic Activity	Gold NPs conjugated with NK cell ligands	[82,83]
Inorganic NPs	B Cells	CD20 Antibodies	Targeted B Cell Depletion	Silica NPs functionalized with CD20 antibodies	[84,85]
	Tumor- Infiltrating Lymphocytes	PD-1 Antibodies	Reinvigoration of TILs	Mesoporous silica NPs with PD-1 antibodies	[86,87]
Metal/Polymeric Hybrid NPs	Dendritic Cells	Toll-like Receptor (TLR) Ligands	Activation of Dendritic Cells	Hybrid NPs with Toll-like receptor ligands	[88,89]
Carbon-Based Nanomaterials	Various	Various	Various Applications in Medicine	Single- and multi-walled carbon nanotubes, graphene oxide, fullerenes, and nanodiamonds for drug delivery and imaging	[90]
Graphene-Based Nanomaterials	Cancer Cells	Hyaluronic Acid	pH-Responsive Drug Delivery	Hyaluronic-acid-decorated graphene oxide nanohybrids for drug delivery	[91]
Superparamagnetic Iron Oxide NPs	Scavenger Receptor	Surface Polymer Coating	Immune Recognition Modulation	Surface-modified iron oxide NPs interacting with scavenger receptors	[92,93]

### 2.2. Modulating Immune Signaling Pathways 

Immunotherapy and the shift towards precise modulation of the immune response have recently received much attention [94]. This development has advanced strategies that previously utilized nanomaterials in immunotherapy to acquire complex tools for controlling immune signaling pathways, which could potentially address previously untreatable diseases [44,79]. Nanomaterials can provoke immunological signaling through physical and chemical cues, penetrating immune pathways and processes associated with chemical carcinogenesis, MAPK signaling, cGMP-PKG signaling, cAMP signaling, and focal adhesions [95]. Similarly, the Wnt/β-catenin pathway not only supports immune responses but also modulates immune response elevation in anti-cancer strategies, thereby fine-tuning the balance between pro- and anti-inflammatory cytokines [96]. Viral proteins remodel innate immune signaling pathways to direct host antiviral responses [10]. In *D. melanogaster*, the cGMP-dependent signaling pathway regulates NF-κB in the immune system. Engineered NPs exhibit a heightened ability to target DCs and promote the development of a tolerogenic phenotype during the induction of immune tolerance [97,98]. Table 5 summarizes the diverse mechanisms and pathways that offer promising targets for the therapeutic regulation and intervention of the immune system.

The intrinsic nature of nanomaterials ensures targeted immune signaling, resulting in desired immunomodulatory effects. Furthermore, a variety of nanomaterials can be utilized to stimulate or suppress the immune system owing to their surface chemistry, which offers a significant potential for immune system modulation [21]. Such intrinsic properties of nanomaterials are related to targeted immune signaling, which achieves desired immunomodulatory effects. The control of immune system activity to achieve the desired immunomodulatory effect largely depends on the surface chemistry of the nanomaterials and their interactions with immune cells and biological molecules [99]. Their purpose may be to stimulate or depress the immune system, which can be manipulated using the surface chemistry of the nanomaterials. For example, surface modifications with ligands or antibodies targeting immune cell receptors allow for selective immune cell activation or inhibition. In addition, the physicochemical properties of nanomaterials, such as their size, shape, and charge, determine their interactions with immune cells and the penetration ability of biological barriers. In general, knowledge of how the physicochemical features of nanomaterials interact with immune system responses is necessary for designing activities in the most effective way for the therapeutic applications of IDDSs [60,100].

Future development includes optimized NPs capable of controlling, sustaining, and releasing antitumor or anticancer drugs as well as bioimaging tracers under high internal physiological hostility [101]. Moreover, nanomaterial-based immunotherapy has recently led to the discovery of a self-driven immune activation method in tumor tissues to prevent adverse immune responses [102]. In contrast, miRNAs modulate key immune signaling pathways by altering the expression of targeted immune system processes and pathways [103]. The successful development of engineered miRNA-based nanomaterials has been instrumental in cancer treatment, specifically in terms of immunomodulation and its effect on the responsiveness of immune cells in cancer immunotherapy [23]. Other potential beneficial effects include specific tumor-killing effects, enhanced immune cell access to crucial metastatic sites, optimized antigen presentation, and induction of idiopathic immune responses [104]. Covalent immobilization of nanomaterials with miRNAs is a promising approach for modulating immune signaling pathways. The utilization of nanomaterials and miRNAs in the formulation of targeted immunotherapies represents an improvement of the limitations of conventional therapy [24,105]. In addition to expanding the therapeutic repertoire, the delivery of miRNAs using nanomaterials is expected to address some of the disadvantages of conventional therapies, such as off-target effects and the development of resistance. Such engineered nanomaterials may precisely target immune signaling pathways to enhance treatment efficacy while limiting side effects, thus offering a new era in immunotherapy with personalized and tailored strategies [106].

### 2.3. Nanomaterials and the Intricate Network of Immune Signaling Pathways

Effective modulation of immune signaling pathways, such as the regulation of cytokines and inhibition of immune checkpoints, can be achieved using nanomaterials including liposomes and polymer-based NPs [99]. These nanomaterials are designed to achieve therapeutic goals by enabling controlled and sustained release of immunomodulatory agents [10,99,107]. It is crucial to note that the controlled release kinetics of these agents are of utmost importance as they determine therapeutic efficacy while minimizing potential adverse effects [3]. Moreover, the surface properties of nanocarriers can be modified after their administration at the target site through functionalization with ligands or antibodies. These modifications facilitate effective interactions with immune cells or selective tissues, thereby inducing an immunological response [107]. The choice of carrier material, such as NPs, significantly affects cargo capacity, release kinetics, and biocompatibility. For instance, mesoporous silica NP- encapsulated interleukin-10 (IL-10) has been utilized to modulate the level of inflammation and associated cascades in autoimmune diseases [108,109]. Furthermore, mesoporous silica NPs were chosen for the encapsulation of IL-10 mainly because of a few significant features that make them particularly suitable for use in controlling inflammation within an autoimmune disease setting. Mesoporous silica NPs have several advantages, such as a high surface area, tunable pore size, biocompatibility, and facile surface functionalization capabilities [63]. These characteristics enable the effective loading and controlled release of IL-10, with maximum cargo capacity and release kinetics tailored to the specific requirements of immune modulation. In addition, mesoporous silica NPs have been proven to have excellent biocompatibility and to significantly decrease unwanted reactions and cytotoxicity, which are the main issues in the case of carrier materials. Their inert nature and stability in physiological environments increases their suitability for in vivo applications, allowing targeted delivery to inflamed sites without compromising systemic safety [110]. 

The development of nanomaterials that regulate immune signaling pathways requires a deep understanding of materials science, pharmacology, and immunology [111]. Nanoparticles coated with programmed cell death protein 1 (PD-1) antibodies can elicit T cell responses upon interaction with tumors, thus promoting antitumor immune activity. PD-1 antibody-coated NPs play a dual role in tumor targeting and immune response regulation. Through interaction with the tumor, PD-1-antibody-coated NPs block the PD-1 receptor on T-cells, which modulates the T-cell inhibitory pathway and, consequently, acts on the immune response. Activated T-cells boost antitumor immune activity against improved tumor recognition and eradication [112]. Material science principles are critical for tailoring the physicochemical properties of nanomaterials to achieve optimal immune modulation. Surface coating, size, and composition dictate the interactions of nanoparticles with immune cells and signaling pathways. Pharmacological advances drive the rational design of nanomaterial-based therapeutics, guiding controlled release kinetics for minimal off-target effects and on-target delivery [113]. In addition, an in-depth understanding of immunological mechanisms is required for targeted immunomodulation with nanomaterials. Such a comprehensive and critical evaluation of preclinical nanotherapeutics may only be achieved with close collaboration among the research community, clinicians, and regulatory bodies so that potential off-target effects and immune sensitization are brought to light [114]. Such interdisciplinary collaboration guarantees safe and effective translation of nanomaterials.

Nanomaterials can modify immune signaling pathways, and close collaboration between researchers, clinicians, and regulatory bodies is essential to ensure rigorous preclinical evaluation to identify any potential off-target effects and sensitization of the immune system [115]. The customization of immunomodulation using nanomaterials to align with the individual profiles of patients will serve as the foundation for personalized immunotherapy. By integrating the principles of precision medicine and systemic immunology interventions, immune responses can be manipulated with high accuracy and precision [116,117]. The convergence of nanotechnology and immunology is a powerful tool for controlling immune responses, thereby overcoming the limitations associated with conventional disease treatments [118]. Triggered release systems offer a promising potential for revolutionizing drug delivery. These systems allow for the precise modulation of immune responses and can be targeted to specific sites of interest, either by activating or inhibiting immune reactions. This signifies the emergence of a new era in precision medicine, providing insights into the future of therapeutics [119,120]. The transition from laboratory discovery to clinical application requires interdisciplinary collaboration, validation of preclinical fundamentals, and committed endeavors to understanding the complexity of immune regulation [44,79]. 

The field of drug delivery has transformed into triggered release systems, as shown in Table 6. These systems have the potential to revolutionize immunomodulatory strategies [77,121]. They control the release of therapeutic agents and allow for the precise modulation of immune responses. Using external or internal signals, these triggered release systems have been used in a new era of precision medicine, reshaping the future of therapeutic interventions [122,123,124].

Triggered release systems are defined by their capacity to react to external or internal signals [125]. External stimuli such as light, temperature, and magnetic fields offer precise and noninvasive triggers. Internal signals depend on the biochemical conditions of the body, including the enzymatic activity, pH, and redox potential. The integration of nanotechnology and immunology has fostered the creation of responsive nanomaterials that selectively release immunomodulatory agents upon encountering specific triggers [126,127,128]. 

The light-induced extraneous response involves the control of the precise amount of light without invasion. Light, as an extraneous trigger, enables precise and noninvasive control. For example, photoresponsive nanomaterials release immunoregulators under specific wavelengths of light, allowing localized calibration of the immune response [129,130]. This allows mild hyperthermia to exploit changes caused by the disease. Therefore, it is crucial to control the extraneous signals to achieve drug release at a specific time in the body. This is particularly important for personalizing immunomodulation and determining an approach to control extraneous signals from drugs [123,131]. 

This mode of action involves utilization of responsive internal cues through biochemical interactions within the body. Specifically, certain enzymes present in the tissue and disease microenvironment catalyze reactions to facilitate the release of drugs in a site-specific manner. This process is effectively achieved by employing nanomaterials bound to enzyme-cleavable linkages, which takes advantage of the abundance of enzymes for drug delivery [132,133]. The difference in pH between healthy and diseased tissues, as illustrated here, plays a crucial role in regulating drug release. Adopting such an approach would enable precise immunomodulation based on the dynamic physiological state of the body [134]. 

Emerging nanomaterial-based systems integrate the responsive properties with immunomodulation. Stimuli-responsive polymers, such as poly(N-isopropyl-acrylamide) (PNIPAM), undergo reversible transitions in response to temperature, enabling controlled drug release. These polymers encapsulate immunomodulatory agents and provide a temperature-triggered release profile [135,136]. Similarly, pH-responsive nanomaterials, such as mesoporous silica NPs and liposomes, selectively release immunomodulatory agents in specific regions upon pH variation. This demonstrates the potential of nanomaterial-based platforms for the precise modulation of the immune response modulation [137,138].

To fully realize this potential, it is crucial to address the challenges associated with achieving the desired release profiles, minimizing off-target effects, and ensuring optimal biocompatibility in triggered-release systems [139]. Further investigation is warranted to explore the interactions between nanomaterials and immunomodulatory agents and their impact on the immune microenvironment [140]. The integration of these materials into immunomodulatory approaches has revolutionized medicine, enabling a new era of personalized treatments.

Controlled spatiotemporal drug delivery has significant potential as an innovative approach for individual treatment with reduced toxicity [99]. This collaborative effort between disciplines has the potential to advance triggered systems, facilitate the translation of laboratory findings into clinical practice, and ultimately revolutionize therapeutic modalities [141].

For example, the improved precision offered by nanotechnology and the enhanced effectiveness of delivery, particularly in drug administration, appears to be highly promising [142]. These advancements are considered the most promising among future nanomaterials, indicating the potential and importance of essential carriers that can align with the complexities of drug delivery and immune modulation (Table 5). This was further supported by an examination of immunomodulation methods using lipid-based NPs, such as liposomes, lipid NPs, and nanoemulsions [143]. The growing array of liposomes, lipid NPs, and nanoemulsions has led to the development of immunomodulatory therapeutic strategies with greater precision and efficacy [144]. Table 7 shows the various roles, features, functions, and applications of the different types of lipidic NPs that orchestrate immune responses.

### 2.4. Mechanisms of Action and Advantages of Lipid-Based Nanomaterials

The efficacy of lipid-based NPs in immunomodulation stems from their physical and chemical characteristics as well as their biocompatibility. These NPs can encapsulate both hydrophilic and hydrophobic immunomodulatory agents, thereby offering a wide range of therapeutic possibilities [150]. Comprised lipid bilayers interact directly with the cell membranes, promoting cellular internalization and intracellular transportation. Additionally, these carriers are biodegradable and have low toxicity, making them ideal for drug delivery and integration into the immune system [151]. 

Lipid-based NPs have unique benefits as vehicles for immunomodulatory drug delivery. Their natural biocompatibility reduces the risk of unintended immune reactions by preventing immune detection [152,153]. They also facilitate controlled and prolonged drug release, which is particularly advantageous in chronic diseases and long-term therapies [154]. These NPs can effectively deliver various immunomodulators such as cytokines, antibodies, and nucleic acids. Notably, they have demonstrated effectiveness in encapsulating siRNAs, enabling the silencing of genes related to immune disorders and showcasing their precision and potential for targeted treatments [155]. Liposomes, a subset of lipid-based NPs, are essential for delivering Toll-like receptor (TLR) agonists, which enhance immune activation and responses to vaccines [60]. Moreover, lipid-based systems have been engineered to enhance the efficacy of immune checkpoint blockade by delivering monoclonal antibodies that target inhibitory receptors. This strategy stimulates potent antitumor immune responses and advances cancer immunotherapy [156]. 

Lipid-based systems are versatile, spanning several therapeutic domains. In the realm of infectious diseases, liposomal formulations encapsulating antimicrobial peptides demonstrate effectiveness in targeted pathogen eradication, showing potential for enhanced treatment modalities [157]. In cancer immunotherapy, lipid-based carriers enhance the efficacy of immune checkpoint inhibitors, counteracting the immunosuppressive effects of tumors, and bolstering anticancer capabilities [158]. However, challenges persist in achieving optimal stability, managing the release kinetics, and scaling up for clinical applications [158]. Understanding the intricate interplay between lipid carriers, immune cells, and the tumor microenvironment is important for advancing this field. Engineered lipid-based systems hold promise for tailoring interventions for specific immune cell subsets, thus enabling more precise immunomodulatory strategies [159]. 

In summary, lipid-based NPs offer immense potential for immunomodulatory drug delivery, presenting exciting prospects for precision medicine [160]. The evolving fields of immunology and nanotechnology are poised to revolutionize therapeutic strategies through the combination of lipid-based carriers and immunomodulators [161]. Polymeric NPs are transformative elements in immunomodulatory drug delivery, and can seamlessly bridge polymer science and immunology. Biodegradable and biocompatible polymers offer a versatile platform for orchestrating immune responses with unparalleled precision [76,112,162,163]. 

### 2.5. Biodegradable and Biocompatible Polymers

This section presents a detailed examination of polymeric NPs in immunomodulation and highlights their potential for future therapeutic interventions (Table 8). The versatility of NPs arises from the diverse range of biodegradable and biocompatible polymers that form their structures. Examples include polylactic acid (PLA), polyglycolic acid (PGA), and poly(lactic-co-glycolic acid) (PLGA). These polymers are known for their natural degradability and safety profiles, forming the basis for NPs that gradually degrade into harmless byproducts, ensuring sustained and safe immunomodulation [9,164]. Additionally, chitosan derived from crustacean shells exhibits unique mucoadhesive properties that are beneficial for mucosal immunomodulation [165].

Polymeric NPs possess distinct functional characteristics, depending on their design. Their size, typically ranging from 50 to 200 nm, significantly affects cellular uptake and biodistribution. Falling within this size range enables NPs to traverse the circulatory pathways while evading immune surveillance. Surface engineering methods, such as incorporating ligands or antibodies, facilitate precise targeting of therapeutic payloads to specific sites [166]. Furthermore, the controlled release of therapeutic agents from polymeric matrices ensures sustained therapeutic effects, reduces dosing frequency, and enhances patient compliance [167].

Polymeric NPs have demonstrated significant impact and transformative potential in various fields of immune modulation. In vaccine development, NPs coated with antigens and constructed from PLGA enhanced immune responses by facilitating sustained antigen presentation and activation of antigen-presenting cells [163]. During the immune checkpoint blockade, polymeric NPs armed with programmed cell death protein 1 (PD-1) antibodies induce antitumor responses by freeing PD-1-expressing T-cells and overcoming tumor-induced immunosuppression. Allergen-loaded NPs facilitate the transition from hypersensitivity to tolerance in allergy immunotherapy. For autoimmune diseases, NPs deliver disease-specific antigens or immunomodulatory agents, restoring immune balance [122]. In gene therapy, polymeric carriers deliver nucleic acids that influence the immune response at a genetic level.

Although polymeric NPs hold great promise, they face challenges, such as achieving ideal release kinetics, maintaining a uniform particle size, and managing batch-to-batch variability. Understanding cellular interactions is critical for their effective use in personalized immunomodulation strategies. Situated at the crossroads between polymer science and immunology, polymeric NPs are cutting-edge tools for delivering immunomodulatory drugs, providing unmatched precision in therapy [109,125,135,163]. This convergence signifies an exciting exchange between disciplines, potentially transforming the landscape of immunomodulation and personalized medicine.

### 2.6. Inorganic NPs for Immunomodulatory Drug Delivery

Inorganic NPs have gained significant recognition as cutting-edge carriers in immunomodulatory drug delivery. These carriers exploit the distinctive properties of materials such as gold, silica, and quantum dots [168,169]. The following section discusses the properties, applications, and challenges associated with inorganic NPs that facilitate precise immune modulation and targeted therapeutic interventions.

Inorganic NPs such as metals, metal oxides, and semiconductors have diverse applications. AuNPs, which are known for their plasmonic properties, are valuable in photothermal therapies and imaging, owing to their strong light absorption and scattering [82]. Silica NPs offer high biocompatibility and adjustable porosity, making them ideal for encapsulating therapeutic agents and facilitating controlled release [53,170]. Quantum dots and nanoscale fluorescent materials enable real-time imaging of immune cells [171]. In immunomodulation, AuNPs are used to induce localized hyperthermia under light, advancing targeted photothermal therapies for tumors and immune cells [82]. Silica nanomaterials are suitable for encapsulating immunomodulatory agents, enabling controlled release owing to their porous structure and large surface area [52,53,170].

These particles can be engineered to target specific immune cells by modifying their surface, allowing them to selectively bind to the cell surface [172,173,174]. For instance, gold NPs can be functionalized with antibodies to enhance T-cell immunity. They also carry molecules that stimulate the immune system and regulate their release [13,49,175]. In contrast, quantum dots enable the real-time tracking of immune cell behavior. Despite their potential, challenges such as achieving a uniform size distribution, maintaining stability, and addressing potential toxicity issues remain [171]. Overall, inorganic NPs represent a promising intersection of nanotechnology and immunomodulation, offering new possibilities for precision medicine [176]. 

## 3. Immunomodulatory Drug Delivery Systems in Cancer Treatment

The integration of nanotechnology and immunotherapy has significantly advanced cancer treatment, particularly for IDDSs. These systems have revolutionized the approach for combating malignancies, offering unmatched precision and efficacy, as shown in Table 7 [177]. Nanomaterial-based systems are instrumental in cancer immunotherapy because they efficiently deliver immunomodulatory agents, including immune checkpoint inhibitors, cytokines, and antigens, to counteract tumors [121]. Leveraging the distinctive characteristics of NPs, these systems surmount physiological barriers and precisely deliver therapeutic payloads to tumor sites, minimize off-target effects, and optimize anticancer responses [178]. 

A groundbreaking development in cancer therapy is the creation of immune checkpoint inhibitors, which empower the immune system to identify and eliminate tumor cells. NP-based systems improve the effectiveness of these inhibitors by transporting them directly into the tumor microenvironment. For instance, gold NPs coated with programmed cell death protein 1 (PD-1) antibodies efficiently hindered inhibitory signals within tumors. This action revitalizes antitumor immune responses and diminishes systemic side effects [179]. 

Cancer vaccines hold promise for educating and empowering the immune system against tumors. However, the challenges associated with antigen presentation and immune recognition have hindered their efficacy [4,147,179,180]. NP-based delivery of vaccines addresses these hurdles by encapsulating tumor antigens within lipid- or polymer-based NPs. This strategy improves antigen uptake by antigen-presenting cells, thereby eliciting strong immune responses against cancer [181]. 

Adoptive cell therapies, notably CAR T-cell therapy, have demonstrated remarkable efficacy against malignancies. NP-based systems can further improve these therapies by encapsulation of immunomodulatory agents. These NPs navigate the intricate immune microenvironment, fostering conditions favorable for antitumor activities and boosting the efficacy of adoptive cell therapies [164,182]. Despite these achievements, challenges remain, such as achieving precise control over drug release, addressing concerns regarding immunogenicity, and comprehending tumor heterogeneity. The intricate orchestration of immune responses necessitates ongoing research to elucidate the interactions between nanomaterial carriers, immune cells, and the dynamic tumor microenvironment.

The future of cancer immunotherapy holds promise for personalized intervention. Advances in genomics, proteomics, and single-cell analysis will enable the integration of nanomaterials with individual tumor immune profiles [124]. Nanomaterials represent a relatively new strategy for cancer immunotherapy, possessing multidimensional value in the targeting of tumor cells, improving the delivery of drugs at the tumor site, and modulating the tumor microenvironment. Such materials can be precisely engineered to target specific antigens on the surface of tumor cells, thereby improving the specificity and potency of immunotherapeutic agents. For instance, gold NPs have excellent photothermal properties that can achieve localized hyperthermia to kill cancer cells, thereby sparing the normal tissues. This is further applicable to drug-carrying liposomes at the nanoscale level for the delivery of chemotherapeutic agents or immunomodulators to tumor sites. By modulating the tumor microenvironment, nanomaterials can be used to enhance immune responses against cancer cells and overcome immunosuppressive barriers. These advances underscore the pivotal role of nanotechnology in reshaping cancer treatment paradigms and improving patient outcomes [183].

Nanomaterials enable the precise modulation of immune responses, offering personalized therapies that address each patient’s unique requirements. IDDSs represent a revolutionary approach to cancer treatment that supports personalized and potent immunotherapy (Table 9). These systems enhance the impact of therapeutic agents by leveraging the synergy between nanotechnology and immunology, thereby leading to a new era in cancer treatment. To address these challenges, harness the potential of nanomaterials, and decipher the complexities of cancer immunity, cancer treatments have shown promise as customized, efficient, and safe therapies. This progress highlights the fusion of scientific and human resilience in cancer treatment.

## 4. Nanomaterial-Based Approaches for Treating Autoimmune Diseases and Chronic Inflammation

Autoimmune diseases challenge the immune system, causing chronic inflammation and tissue damage due to self-targeting dysregulated immune responses. Because the immune system is intricately involved in the pathogenesis and immune regulation of autoimmune diseases, this presents an opportunity for the most efficient nanomaterials to act as a solution for regulating the immune response by precisely targeting aberrant immune pathways and drug delivery of immunomodulatory agents in autoimmunity-affected tissues [184]. It is feasible to design nanomaterials that selectively interact with the immune cells or tissues responsible for autoimmune pathology to restore immune homeostasis and dampen adverse inflammatory responses. For example, polymeric NPs can carry either immunosuppressive drugs or antigens, allowing targeted delivery to the site of inflammation, with minimal systemic side effects. Similarly, lipid NP vesicles such as exosomes may be utilized to transport regulatory miRNAs or peptides to modulate immune cells and suppress autoimmune reactions. Evidence of nanomaterial-based therapies in both preclinical and clinical applications has shown promise and has been successful in a range of autoimmune disorders including RA, multiple sclerosis (MS), and systemic lupus erythematosus [185,186]. The aim is to exploit the distinct properties of nanomaterials in the development of new approaches for personalized and precision medicine in the management of autoimmune diseases, which will also improve the outcomes and quality of life of patients [186].

The combination of nanotechnology and immunology has led to innovative precision medical strategies to address autoimmune diseases and chronic inflammation. Nanomaterial-based systems, including lipids, polymers, and inorganic NPs, hold significant promise for revolutionizing the treatment of these conditions. These systems, such as liposomes, polymeric NPs, and inorganic NPs, are meticulously designed to efficiently encapsulate immunomodulatory agents [173]. By exploiting the nanoscale, these carriers can selectively accumulate at sites of inflammation, thereby diminishing the likelihood of systemic side effects, while enhancing the effectiveness of treatment. Drug delivery systems with immunomodulatory capabilities have been designed to target specific pathways and immune cell populations relevant to these diseases. For instance, in RA, NPs containing anti-inflammatory cytokines, such as IL-10, have demonstrated efficacy in reducing joint inflammation [13,45,72]. 

Scientists have engineered nanocarriers to ferry disease-altering medications through the blood–brain barrier (BBB) in MS, thereby mitigating inflammation within the central nervous system. Furthermore, NPs infused with anti-inflammatory substances have been used to modulate immune reactions in patients with inflammatory bowel disease (IBD) patients. The burgeoning potential of nanomaterial-driven systems in tackling autoimmune and inflammatory ailments has been bolstered by the expanding body of preclinical and clinical investigations [173,187]. Experimental models have demonstrated the capacity of nanocarriers to impede disease progression, limit tissue damage, and reinforce the immune equilibrium. Phase II clinical assessments have underscored the safety and effectiveness of nanomaterial-centric interventions for afflictions such as psoriasis, asthma, and lupus [188]. Despite the undeniable promise of nanomaterial-based methodologies for addressing these ailments, obstacles persist [189]. These findings underscore the potential of nanotechnology to drive a paradigm shift in approaches used to treat chronic inflammatory diseases. Although such advancements hold great promise, the translation of nanomaterial-based methodologies to clinical applications is limited [190]. It is important to emphasize the necessity of optimal biocompatibility, scalable and reproducible manufacturing processes, and acceptable long-term safety profiles. Furthermore, regulatory approval, protocol standardization, and cost-effectiveness are crucial factors that must be addressed to facilitate the widespread use of nanotherapeutics and improve outcomes in various patient populations [113]. As such, a major emphasis should be placed on strict preclinical scrutiny, careful design of clinical trials, and post-marketing surveillance to minimize risk and ensure the safe and effective deployment of nanomaterial-based interventions [114]. Further improvement in the knowledge related to pathophysiological diseases and the interaction of the host with nanomaterials will aid in designing appropriate nanotherapeutics for specific patients. These personalized medical modalities include biomarker profiling and patient stratification, which improve treatment efficacy and reduce negative effects [191]. In conclusion, nanomaterial-based approaches are promising for treating chronic inflammatory diseases. However, there remains a need to overcome overall impediments through coherent cooperation and interdisciplinary collaboration. Overcoming these challenges would, therefore, pave the way for nanotechnology in disease management for better patient outcomes.

The intricate relationship between nanomaterials, therapeutic agents, and the immune system requires a thorough understanding. Achieving targeted drug delivery, optimizing release profiles, and ensuring long-term safety require meticulous research and consideration. As the field progresses, the future of personalized management of autoimmune and inflammatory diseases relies on integrating biomarker identification, immune profiling, and tailored nanomaterial-based strategies according to disease phenotypes and individual immune signatures [192]. Nanomaterial-based approaches have revolutionized disease management by combining nanotechnology precision with immunological complexity. These strategies finely tune immune responses and have been proven to be accurate and effective in treating autoimmune diseases and chronic inflammation, thus marking the beginning of a new era of immune modulation [193]. With the emergence of nanomaterial-based IDDSs in precision medicine, meticulous safety considerations are imperative. The intricate interaction between nanomaterials and immune responses requires thorough evaluation to ensure the safety and efficacy of these interventions [194]. The advancement of safe immunomodulatory drug delivery systems relies on the biocompatibility of the nanomaterials. Achieving this requires NPs to navigate physiological barriers, interact with immune cells, and regulate drug dispersal, while minimizing potential adverse effects [195]. When assessing the potential toxicity of NPs, critical factors such as size, surface chemistry, charge, and composition must be carefully considered because these factors significantly affect cellular uptake, immune response, and systemic distribution [196]. 

Comprehensive safety assessments are crucial for the successful translation of nanomaterial-based systems from the laboratory to clinical settings. In vitro studies are essential for understanding cellular reactions and cytotoxicity as they provide initial data on the compatibility of nanomaterials with immune cells [197]. Animal models have been pivotal in evaluating the toxicological effects of various NPs, underscoring the need for improved physicochemical assays to accurately assess exposure risks and predict their behavior and therapeutic potential in vivo [198]. Animal models have been particularly useful for studying the toxic effects of dendrimers, silver NPs, gold NPs, and carbon nanotubes [199]. 

Enhancing the safety of nanomaterial-based IDDSs requires a multi-faceted approach. This involves designing NPs with tailored physicochemical characteristics to minimize immune recognition and cellular uptake [200]. Surface modifications using biocompatible polymers or stealth coatings can effectively reduce the opsonization and immune clearance. The selection of biodegradable materials ensures that NPs are metabolized into harmless byproducts, thereby minimizing the potential long-term effects [201]. 

Personalized safety assessments are essential for developing nanomaterial-based immunomodulatory systems. Recognizing unique variations in immune responses, genetic profiles, and underlying health conditions emphasizes the need for tailored intervention [202]. Advanced techniques, such as organ-on-a-chip models and patient-derived cell cultures, provide sophisticated platforms for personalized safety assessment and refine therapeutic strategies to achieve optimal safety and effectiveness [203]. 

The transition from research exploration to the clinical application of nanomaterial-based systems requires adherence to comprehensive regulatory guidelines and standardized procedures. Regulatory bodies scrutinize safety data, necessitating robust evidence of biocompatibility, pharmacokinetics, and toxicity, as summarized in Table 10. Standardized protocols governing nanomaterial synthesis, characterization, and safety assessment promote consistency in reporting, transparency enhancement, and credibility across studies [85,197]. Innovations in nanomaterial-based IDDSs must prioritize safety and draw lessons from past experiences in future research. Collaboration among scientists, clinicians, and regulatory authorities is key to balancing scientific advancements with patient safety. Nanomaterials offer promising potential for therapeutic advancements, ensuring patient safety through comprehensive evaluation, personalized methodologies, and regulatory adherence [200]. Navigating the complexities of nanotechnology demonstrates a commitment to establishing a future in which innovative and patient-centered immunomodulatory treatments can become a reality.

## 5. Conclusions and Future Directions

In medicine, tailored and precise techniques are gaining traction with the combination of nanotechnology and immunology, resulting in the development of IDDSs. This review focuses on these systems and investigates their transformational potential in immunotherapy and disease treatment. We summarize the major discoveries, evaluate the possibilities of nanomaterial-based systems, and provide insights into future research. 

IDDSs that use nanomaterials provide promising opportunities to modify the immune responses to various illnesses. By combining nanotechnology with immunology, these approaches allow for targeted administration of immunomodulatory drugs to specific immune cells and inflamed areas. This study focuses on how nanomaterials can improve immune checkpoint inhibitors, cancer vaccines, and adoptive cell therapies, resulting in more effective treatments tailored to specific patients. However, further refinement are required. Optimization of NP properties, surface qualities, and targeting abilities is critical for improving their interactions with immune cells.

The use of personalized therapies based on patient-specific immune markers, coupled with the integration of artificial intelligence into material design, has the potential to expedite the identification of promising candidates for clinical application. The introduction of nanomaterial-based systems into clinical practice necessitates careful management of regulatory complications, safety issues, and scalability. Achieving a balance between scientific innovation and regulatory compliance is therefore critical. Thorough safety reviews, toxicity assessments, and rigorous clinical trials are required to ensure patient safety and therapeutic efficacy. Acceleration of the clinical translation of nanomaterial-based immunotherapies will require collaborative approaches, early engagement with regulatory bodies, optimized trial protocols, engagement with patients, increased investment, international cooperation, risk management, strategic partnerships, and real-world evidence. 

Advances in nanotechnology promise to improve drug delivery to specific immune cells, thereby extending its application in immunomodulation. Personalized therapy, which is driven by patients’ immunological profiles and AI, enables rapid clinical implementation. Collaboration between scientists and clinicians has accelerated translation efforts. Tailored regulations are critical for broadening therapeutic applications of nanomaterial-based immunomodulation. Exploring molecular-level immune responses requires precise manipulation using sophisticated nanomaterial design. Overcoming obstacles, such as scalability, consistency, and safety, is critical for effective clinical translation. The focus on patient-centered outcomes prioritizes treatment effectiveness, quality of life, and adherence to nanomaterial-based therapeutics. The anticipated worldwide collaboration will accelerate advances in immunomodulation and medication delivery, thereby boosting the creativity and efficacy of treatment approaches. Nanomaterial-based immunotherapy has shown promise in preclinical research and has moved into early clinical trials, with ongoing efforts focused on targeted delivery and controlled release to specific cells. However, this strategy is not widely applied in clinical practice yet, and further development is required to overcome current challenges.

## Figures and Tables

**Table 1:** Comparison of advantages and disadvantages of different types of nanomaterials for biomedical applications.

NPs Type	Advantages	Disadvantage
Organic Dye NPs	High biocompatibility and low toxicity	Lower payload than for other nanomaterials
	Versatile surface chemistry for functionalization and targeting	Prone to photodegradation and photobleaching
	Strong optical properties for imaging and phototherapy applications	Challenges in achieving long-term stability in physiological environments
Silica NPs	Rigid, tunable, and porous structure for high drug loading and controlled delivery	Concerns regarding long-term biodegradation and persistence
	Good biocompatibility and low immunogenicity	Poor encapsulation of hydrophobic drugs
	Amenable to surface modifications for targeted delivery and imaging	Promotion of nonspecific interaction with biological components
Metal NPs	Unique physicochemical properties, like plasmonic and magnetic responsiveness	Biocompatibility concerns and potential toxicity issues, especially with heavy metal-based NPs
	Multimodal imaging and the combination of photothermal/photodynamic therapy is possible	Challenges: precise control over size, shape, and surface properties
	Easy synthesis and surface functionalization	Low stability and predisposition to aggregation in biological environments

**Table 2:** Overview of immunotherapy paradigms showing the potential mechanism of action, clinical efficacy, and side effects on the immune system.

Immunotherapy Paradigm	Mechanism of Action	Clinical Efficacy	Side Effects
Immune Checkpoint Inhibitors	Binding of inhibitory checkpoints (e.g., PD-1/PD-L1, CTLA-4) to liberate antitumor immune responses	Proved effective in numerous malignancies, including melanoma, non-small cell lung cancer, and renal cell carcinoma	Immune-related adverse events, such as dermatitis, colitis, and pneumonitis
Chimeric Antigen Receptor (CAR) T cell Therapy	Genetic manipulation of patient’s own T cells with the gene for chimeric antigen receptors recognizing tumor-specific antigens	Excellent responses were observed in hematologic cancers, particularly in relapsed/refractory B-cell acute lymphoblastic leukemia and non-Hodgkin’s lymphoma	Cytokine release syndrome, neurotoxicity, and on-target/off-tumor effects
Cancer Vaccines	Stimulation of specific immune responses against tumor-associated antigens by vaccination	Limited success in solid tumors, with some exceptions in prostate cancer (Sipuleucel-T) and melanoma (T-VEC)	Local reactions at the site of injection, flu-like symptoms, and autoimmune reactions
Adoptive Cell Transfer	Infusion of ex vivo expanded autologous tumor-infiltrating lymphocytes (TILs) or genetically engineered T cells	Impressive responses were seen in melanoma and selected solid tumors	Cytokine release syndrome and graft-versus-host disease (with allogeneic T cells)
Oncolytic Viral Therapy	Selective replication of viruses in tumor cells and their lysis, leading to immune activation	Preliminary results in clinical trials for malignancies such as melanoma, glioblastoma, and advanced solid tumors	Local inflammation at the tumor site, flu-like symptoms, potential for viral shedding
Checkpoint Inhibitor Therapy in Combination with Chemotherapy	Concurrent delivery of immune checkpoint inhibitors with conventional chemotherapy agents to augment antitumor immune responses	Improved overall survival and progression-free survival in multiple cancers, including lung cancer, triple-negative breast cancer, and bladder cancer	Enhanced potential for adverse events related to chemotherapy, including cytopenia, nausea, and alopecia; potential for immune-related adverse events in an additive manner
Bispecific Antibodies	Act as the bridge between the tumor cells and immune effector cells via dual binding of CD3-positive T cells and tumor-specific antigens	Proven clinical benefit in hematological malignancies, especially acute lymphoblastic leukemia and multiple myeloma; evolving evidence in solid tumors	Cytokine release syndrome; potential for off-tumor toxicities
Modulating the Tumor Micro- environment	Alteration of the tumor microenvironment to potentiate immune responses to the tumor	Preliminary evidence of potential efficacy in overcoming immunosuppressive barriers in the tumor microenvironment and enhancing responses to immunotherapy	Risk of exacerbating autoimmune reactions; potential for off-target effects on normal tissues
Dendritic Cell Vaccines	Usage of dendritic cells loaded with tumor antigens to induce specific immune responses	Some clinical success to date but ongoing research to optimize the strategy of dendritic cell vaccines	Localized reactions at the injection site, flu-like symptoms, and possibility for autoimmune reactions

**Table 3:** Strengths, weaknesses, opportunities, and threats related to the application of nanomaterials in immunotherapy.

Strengths	Weaknesses	Opportunities	Threats
Precision Targeting: Nanomaterials offer precise delivery of immunomodulatory agents, potentially reducing treatment frequency.	Complex Design: Design esign intricacies extend to maintaining stability and consistency during storage and transportation.	Personalized Medicine: Advancements in immune profiling enable tailored treatments and identification of individual immune signatures.	Regulatory Hurdles: Strict regulations may lead to delays in clinical translation and commercialization, necessitating comprehensive safety assessments.
Enhanced Efficacy: Nanocarriers enable controlled release, optimizing immune modulation and treatment outcomes.	Potential Toxicity: Thorough safety assessments must account for potential biodegradability issues and tissue clearance of nanomaterials.	Multidisciplinary Collaboration: The collaboration of nanotechnology and immunology experts generates innovative solutions and strategies for immunotherapy.	Immunogenicity: the risk of immune reactions triggered by nanomaterials could impact both the efficacy and safety of treatment.
Diverse Applications: Nanomaterials find application in cancer therapy, autoimmune diseases, and inflammation, expanding the scope of precision medicine.	Manufacturing Challenges: Scaling up nanomaterial production while ensuring consistent quality remains a challenge, affecting widespread adoption.	Drug Combination Therapy: Nanocarriers enable synergistic effects and novel combinations, offering avenues for enhanced treatment strategies.	Resistance Development: Prolonged usage of nanomaterials might lead to immune cell resistance, potentially reducing therapeutic effectiveness.
Immune Cell Modulation: Nanomaterials allow targeted modulation of immune cells, promoting precise immune responses and homeostasis.	Biodistribution Variability: Variability in nanoparticle distribution among individuals could impact treatment outcomes and response rates.	Minimized Side Effects: Accurate targeting reduces off-target effects, limiting damage to healthy tissues and improving patient tolerance.	Long-term Effects: The potential accumulation of nanomaterials in tissues might lead to unforeseen long-term effects on health and the environment.

**Table 5:** This table provides an overview of nanomaterials involved in the regulation of immune signaling pathways.

Nanomaterial	Immune Signaling Pathway	Mechanism of Action	Applications	Challenges and Considerations
Liposomes	Cytokine Modulation	Encapsulation and controlled release of cytokines	Cancer immunotherapy, Autoimmune disorders	Variability in release kinetics, the potential for immunogenicity
Gold NPs	Immune Checkpoint Blockade	Surface functionalization with checkpoint inhibitors	Cancer immunotherapy	Optimal dosage, potential off-target effects
Lipid NPs	Cytokine Modulation	siRNA delivery for cytokine modulation	Inflammatory diseases, Vaccination	Intracellular delivery efficiency, stability
Polymeric NPs	Immune Checkpoint Blockade	Controlled release of checkpoint inhibitors	Cancer immunotherapy	Long-term biocompatibility, controlled release optimization
Quantum Dots	Cytokine Modulation	Photo stimulation-induced cytokine production	Immunomodulation	Phototoxicity, long-term effects
Carbon Nanotubes	Immune Checkpoint Blockade	Functionalization for checkpoint inhibition	Cancer immunotherapy	Biodistribution, biodegradation
Micelles	Toll-like Receptor Modulation	Encapsulation of TLR agonists	Vaccine adjuvants, Immunotherapy	Stability in physiological conditions, potential for TLR activation
Magnetic NPs	Macrophage Activation	Magnetic targeting of macrophages	Drug delivery, Immunotherapy	Optimal magnetic field strength, potential for non-specific binding
DNA NPs	Antigen Presentation	Display of antigens on DNA scaffolds	Vaccines, Immunotherapy	Immunogenicity, stability in biological environments
Protein NPs	Immune Cell Activation	Presentation of immunostimulatory proteins	Cancer immunotherapy, Vaccines	Protein stability, the potential for immune recognition
Hybrid NPs	Dual Modulation	Combination of different immune modulation strategies	Autoimmune disorders, Cancer immunotherapy	Optimization of hybrid composition and properties, potential for off-target effects

**Table 6:** Stimuli-responsive nanomaterials developed for precise immunomodulation can be activated upon demand for immunomodulatory purposes.

Stimulus	Trigger Mechanism	Nanomaterials and Systems	Immunomodulation Applications	Advancements and Challenges
Light	Photothermal Effects	Gold NPs, Carbon Nanotubes	Cancer immunotherapy (e.g., PD-L1 targeting)	Enhanced tissue penetration, e.g., NIR-II
	Photochemical Reactions	Liposomes (encapsulating photosensitizers), Quantum Dots	Photodynamic immunotherapy (e.g., ROS induction)	Spatiotemporal precision, photochemical stability
Temperature	Thermosensitive Polymers	Lipid-based NPs (e.g., liposomes)	Fever-range activation for controlled inflammation	External control, systemic effects
pH	pH-Responsive Polymers	Polymeric NPs (e.g., micelles)	pH-triggered drug delivery in tumor microenvironment	pH-responsive release kinetics, stability
	pH-Activated Nanomaterials	Mesoporous Silica NPs	pH-dependent cytokine modulation	pH range compatibility, controlled release
Enzymatic Activity	Enzyme-Responsive Systems	Lipid-based Nanovesicles (e.g., exosomes)	Wound healing, enzyme-targeted immunomodulation	Enzyme specificity, stability
Redox Potential	Redox-Responsive Nanomaterials	Nanogels, Liposomes	Oxidative stress modulation in autoimmune disorders	Intracellular release, bioavailability
Radiological	Radiation	Radioactive NPs	Cancer immunotherapy, Tumor ablation	Targeted delivery to tumors, radiation dose optimization
Ultrasound	Acoustic Waves	Ultrasound Contrast Agents, Microbubbles	Drug delivery, Immunomodulation	Non-invasive, targeted delivery, safety concerns
Magnetic Fields	Magnetic Forces	Magnetic NPs	Immunomagnetic targeting, Drug delivery	Targeted delivery, magnetic field strength optimization
Electric Fields	Electrical Signals	Electroconductive Nanomaterials	Neuromodulation, Tissue regeneration	Precise control, Biocompatibility
Mechanical Strain	Physical Stress	Nanocomposite Hydrogels, NPs	Tissue engineering, Regenerative medicine	Mechanical properties, biodegradability

**Table 7:** Characteristics, mechanisms, advantages, and applications of lipid-based nanomaterials in immunomodulation for precise drug delivery.

Nanomaterial	Mechanisms of Action and Advantages	Examples of Systems and Therapeutic Applications
Liposomes	Encapsulation: efficiently encapsulate hydrophilic and hydrophobic drugs. Biocompatibility: low toxicity and immunogenicity. Surface modification for site-specific drug delivery.	Encapsulation of cytokines (e.g., IL-2) for cancer immunotherapy. Co-delivery of tumor antigens and adjuvants for cancer vaccines [145].
Lipid NPs	Increased Drug Loading Capacity Sustained Release: achieve controlled and prolonged release patterns. Cellular Uptake: facilitate efficient absorption by immune cells.	Lipid NPs loaded with siRNA targeting TNF-α for the treatment of inflammatory diseases. Lipid NPs loaded with curcumin for immune modulation in autoimmune disorders [146,147,148].
Nanoemulsions	Nano-sized droplets, which exhibit stability and compatibility with the human body, possess various advantages in the field of biomedical research. One significant characteristic of these droplets is their versatility, as they can be employed for a wide range of administration techniques. Furthermore, the immunomodulatory properties of nano-sized droplets hold great potential in activating immune responses.	Enhancing vaccine responses through application of oil-in-water nanoemulsions as adjuvants. Skin cancer immunotherapy: topical delivery of resiquimod-loaded nanoemulsions [149].

**Table 8:** Polymeric NPs formulated to deliver immunomodulatory drugs.

Polymeric NPs	Design Considerations	Case Studies and Efficacy in Immunomodulation
Biodegradable and biocompatible polymers	Biodegradability: select polymers with controlled degradation into non-toxic byproducts. Biocompatibility: minimize immunogenicity for enhanced safety.	PLGA NPs co-delivering tumor antigens and adjuvants, boosting cancer vaccine responses. Chitosan-based NPs as tolerogenic carriers in autoimmune disease management.
Design considerations	Size Optimization: tailor nanoparticle size for efficient cellular uptake and lymphatic drainage, influencing immune cell interaction. Surface Modification: functionalize surfaces for targeted delivery to specific immune cell populations. Controlled Release: implement sustained release strategies for prolonged immunomodulation.	PEGylated polymeric NPs with tailored size delivering miRNA for inflammation regulation in colitis. Zwitterionic polymer-coated NPs achieving controlled release of checkpoint inhibitors, enhancing anticancer immune response.
Case studies and efficacy	PLGA-based NPs loaded with immune-modulating agents exhibit improved tumor regression in murine models.	PCL NPs loaded with anti-inflammatory cytokines showcase reduced joint inflammation in arthritis models. pH-responsive polymeric NPs effectively release immunomodulatory drugs in response to the tumor microenvironment.

**Table 9:** Lipid-based, polymer-based, and other types of NPs, focusing on functionalized surfaces for targeted drug delivery in cancer treatment within the tumor microenvironment (TME).

Cancer Immunotherapy	Nanomaterial-Based Systems	Case Studies in Efficacy
Nanomaterial-Based Systems	Liposomes, lipid NPs, and polymer-based NPs engineered for targeted drug delivery and sustained release. Surface functionalization with ligands for enhanced tumor cell targeting and immune cell interaction.	Co-delivery of PD-1 inhibitors and tumor antigens via liposomes, achieving synergistic checkpoint blockade and antigen presentation. PLGA-based cancer vaccines inducing robust immune responses against specific tumor antigens. Gold NPs conjugated with CAR-T-cell-targeting ligands for improved tumor penetration.
Case Studies on Efficacy	Lipid-based NPs co-encapsulating checkpoint inhibitors and tumor antigens for potent antitumor immune responses.	Nanoemulsions delivering immune adjuvants, enhancing DC activation and immune memory in cancer vaccines. Polymeric NPs prolong CAR T-cell persistence and enable sustained tumor surveillance.
Challenges and Future Directions	Optimizing nanoparticle properties for efficient tumor accumulation and controlled drug release. Overcoming immunosuppressive tumor microenvironment barriers for effective immune activation.	Nanoparticle-mediated combinatorial strategies, leveraging synergies between immunomodulators and conventional therapies. Personalized approaches tailoring nanoparticle formulations to individual patient profiles for optimized outcomes.

**Table 10:** Comprehensive information regarding biocompatibility and potential toxicity factors must be considered during the development of IDDSs.

Biocompatibility and Toxicity	Nanomaterial Evaluation
Biocompatibility and toxicity considerations	Conducting a thorough examination of how nanomaterials affect immune cells, cytokine levels, and immune activation pathways. Investigating the immunogenicity and ability to induce adaptive immune responses of nanomaterials. This analysis should include assessing the compatibility of nanomaterials with various administration routes to minimize adverse effects.
Nanomaterial evaluation	Performing in vitro cell culture studies with relevant immune cell types to assess biocompatibility, immune response, and potential cytotoxicity. Utilizing suitable animal models for in vivo investigations to analyze biodistribution, potential organ-specific toxicity, and immune reactions. Incorporating advanced techniques like intravital imaging and single-cell analysis to provide real-time insights into the behavior of nanomaterials and their interactions with the immune system.

## Data Availability

No data were generated during the preparation of this manuscript.
